# Supplementary material for: Causal Relationship between Aging and Anorexia Nervosa: A White-Matter-Microstructure-Mediated Mendelian Randomization Analysis
Source: Biomedicines. 2024 Aug 16;12(8):1874. doi: 10.3390/biomedicines12081874 (PMC11351342; doi:10.3390/biomedicines12081874)
Supplement: Supplementary file 1 [file biomedicines-12-01874-s001.zip › Supplementary Figure/Figure S9/Leaveoneout plot of MR analysis for ubm-b-1514 to AN.pdf]

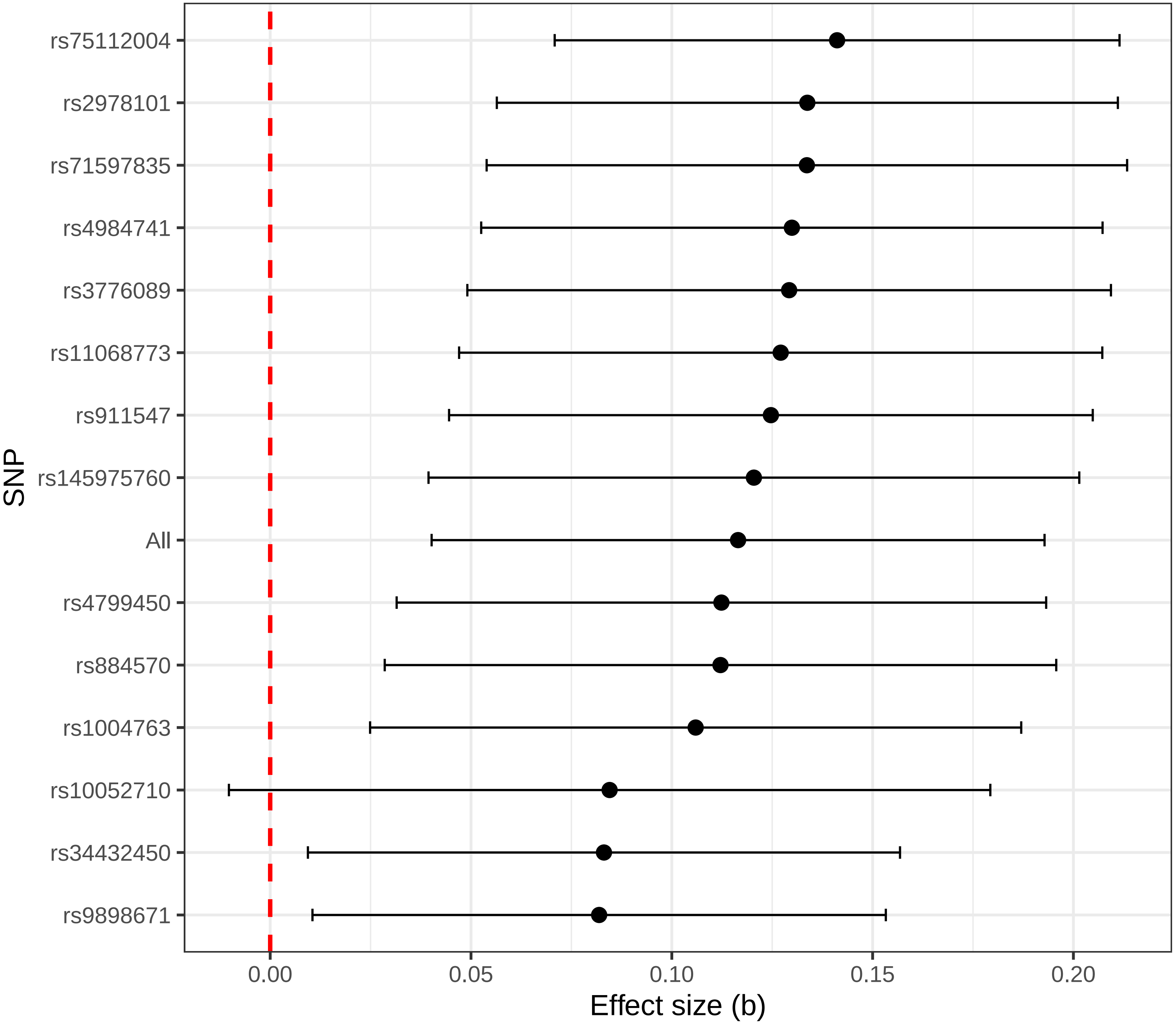
rs75112004
rs2978101
rs71597835
rs4984741
rs3776089
rs11068773
rs911547
rs145975760
All
rs4799450
rs884570
rs1004763
rs10052710
rs34432450
rs9898671
SNP
0.00
0.05
0.10
0.15
0.20
Effect size (b)
